# Draft genome sequence of *Fusicladium effusum*, cause of pecan scab

**DOI:** 10.1186/s40793-016-0161-y

**Published:** 2016-06-03

**Authors:** Clive H. Bock, Chunxian Chen, Fahong Yu, Katherine L. Stevenson, Bruce W. Wood

**Affiliations:** Southeastern Fruit and Tree Nut Research Lab, USDA, Agricultural Research Service, 21 Dunbar Road, Byron, GA 31008 USA; Interdisciplinary Center for Biotechnology Research, University of Florida, 2033 Mowry Road, Gainesville, FL 32610 USA; Department of Plant Pathology, University of Georgia, 2360 Rainwater Rd., Tifton, GA 31793 USA

**Keywords:** *Fusicladium effusum*, Venturiacae, Fungal pathogen, Fungicide resistance, Genetic diversity, Pecan, Pecan scab

## Abstract

Pecan scab, caused by the plant pathogenic fungus *Fusicladium effusum*, is the most destructive disease of pecan, an important specialty crop cultivated in several regions of the world. Only a few members of the family Venturiaceae (in which the pathogen resides) have been reported sequenced. We report the first draft genome sequence (40.6 Mb) of an isolate *F. effusum* collected from a pecan tree (cv. Desirable) in central Georgia, in the US. The genome sequence described will be a useful resource for research of the biology and ecology of the pathogen, coevolution with the pecan host, characterization of genes of interest, and development of markers for studies of genetic diversity, genotyping and phylogenetic analysis. The annotation of the genome is described and a phylogenetic analysis is presented.

## Introduction

**Fig. 1:**
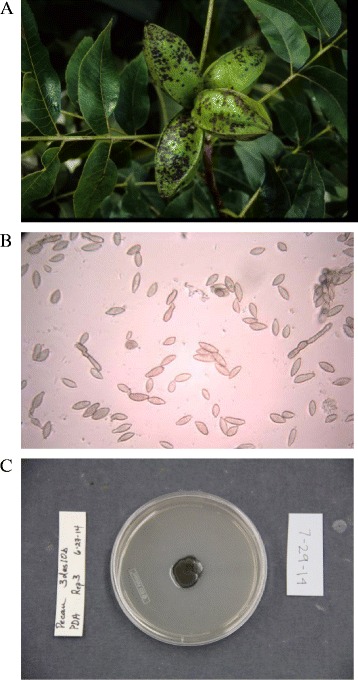
**a** Scab symptoms on pecan fruit, caused by *Fusicladium effusum*. **b** Conidia of *F. effsum* (400×). **c** A 2-week old colony of *F. effsum* growing on PDA

The pecan scab fungus (*Fusicladium effusum* [G. Winter]) is an economically important pathogen of pecan (*Carya illinoinensis* [Wangenh]. K. Koch), on account of its impact on yield and quality of valuable nutmeats [1–3]. Typical lesions resulting from infection by the pathogen are small (generally 1–5 mm) blackish and necrotic, forming on leaves, fruit and shoots (Fig. 1a) [4]. *F. effusum* overwinters as stromata in the lesions on twigs and old shucks in the pecan tree, producing conidia in the spring as temperatures rise, which infect developing leaves and fruit [5]. Both rain and wind play a role in dispersal of conidia, and surface moisture is required for infection [6, 7]. It is a polycyclic disease, with as little as 7–9 days between infection and sporulation [7], allowing epidemics to develop rapidly when rain is frequent during the spring and summer [8].

Although pecan is native to the US, it is grown commercially elsewhere and the pathogen now occurs not only in the US, but in South America, and South Africa as well [4]. *F. effusum* reproduces asexually through production of conidia [6], it is pathogenically diverse [9–12], affecting many different cultivars, and has a history of adapting to novel sources of host resistance [2]. Preliminary molecular studies suggested it is a genetically diverse organism [13, 14], yet no sexual stage has been identified for this fungus. But as the genetic basis of resistance and virulence has not been characterized, progress in breeding resistance is severely hampered. Furthermore, *F. effusum* has developed insensitivity to several classes of fungicide that are used to manage the pathogen [15].

Some related members of the Class Dothidiomycetes, and the family Venturiacae, in which *F. effusum* resides, have been sequenced [16–21], including *Venturia inaequalis* (cause of apple scab) and *V. pirina* (cause of pear scab). These organisms can have impact on plant health, and in some cases animal and human health. These fungal genome sequences provide an opportunity to apply novel genomic and biotechnological tools to develop new solutions to the issues resulting from the interaction of these organisms with their hosts.

With respect to pecan scab, a more thorough understanding of the pathogen and its genetics are needed to develop durable resistance in the pecan host. Sequencing the genome of *F. effusum* will provide a valuable resource to use for identifying various genes of interest, such as those involved in fungicide resistance, those involved in host recognition, mating-type genes, and identification of microsatellites to study genetic diversity (or as markers for other purposes). We describe the first draft genome sequence of *F. effusum*, the characteristics of annotation, and provide a phylogenetic analysis of the taxonomy of the pathogen. The genome sequence will provide an opportunity for new research to gain insight into fundamental aspects of this economically important disease of pecan.

## Organism information

### Classification and features

The sequenced strain of *F. effusum* was isolated from a scab-infected pecan fruit in a 28-y-old tree (cultivar ‘Desirable’) in July 2010 at the USDA-ARS-SEFTNRL, Byron, Georgia, US (Table 1). Conidia (Fig. 1b) of *F. effusum* were scraped from a single lesion on the fruit using a scalpel, and a dilute spore solution prepared in sterile distilled water. Multiple 0.1 μL aliquots were spread on the surface of replicate water agar plates (WA, amended with lactic acid [0.50 mL/L], streptomycin [0.20 g/L], tetracycline [0.05 g/L] and chloramphenicol [0.05 g/L]). Plates were incubated at 27 °C for 24 h under fluorescent light on a 12/12 h day/night cycle. A single germinated spore of *F. effusum* was excised on an agar plug using a scalpel under a microscope (50×), and was transferred to antibiotic-amended potato dextrose agar (PDA [22], amended as for WA) (Fig. 1c).

**Table 1:** Classification and general features of *Fusicladium effusum* designation [37]

MIGS ID	Property	Term	Evidence code^a^
	Classification	Domain *Fungi*	TAS [4, 24]
		Phylum *Ascomycota*	TAS [4, 24]
		Class *Dothidiomycetes*	TAS [4, 24]
		Order *Pleosporales*	TAS [4, 24]
		Family *Venturiaceae*	TAS [4, 24]
		Genus *Fusicladium*	TAS [4, 24]
		Species *Fusicladium effusum*	TAS [4, 24]
	Gram stain	n/a	n/a
	Cell shape	Mycelium with septae	TAS [4, 24]
	Motility	Non-motile	TAS [4, 24]
	Sporulation	Conidia on conidiophores	TAS [4, 6]
	Temperature range	Mesophilic (10–35 °C)	TAS [7]
	Optimum temperature	15–25 °C	TAS [7]
	pH range; Optimum	Not reported	n/a
	Carbon source	Not reported	n/a
MIGS-6	Habitat	Arboreal	TAS [4]
MIGS-22	Oxygen requirement	Aerobic	TAS [4, 24]
MIGS-15	Biotic relationship	Free living	TAS [4]
MIGS-14	Pathogenicity	Pathogenic	TAS [4]
MIGS-4	Geographic location	Byron, Georgia, USA	TAS
MIGS-5	Sample collection	July 2010	TAS
MIGS-4.1	Latitude	32.652° N	TAS
MIGS-4.2	Longitude	83.739 ° W	TAS
MIGS-4.4	Altitude	156 m	TAS

^a^Evidence codes - *IDA* inferred from direct assay, *TAS* traceable author statement (i.e., a direct report exists in the literature), *NAS* non-traceable author statement (i.e., not directly observed for the living, isolated sample, but based on a generally accepted property for the species, or anecdotal evidence). Evidence codes as for the Gene Ontology project [37]

**Fig. 2:**
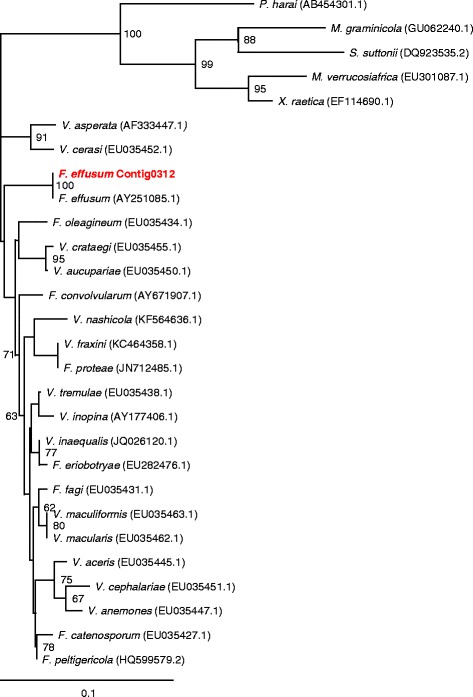
The phylogenetic position of *Fusicladium effusum* in comparison with other related fungal species. The tree was developed based on the 18S rRNA gene of the sequenced isolate of *F. effusum*, an accession of the 18S rRNA gene of another *F. effusum* isolate, and accessions of other members of the family Venturiaceae (genera *Fusicladium* and *Venturia*) and an outgroup with representatives from other Ascomycota from the class Dothidiomyectes (*Phyllosticta harai*, *Staniwardia suttonii*, *Mycosphaerella graminicola* and *M. verrucosiafricana*). The sequence data were subjected to phylogenetic analysis using CLUSTALX2 [25] and MEGA5 [38] to construct a nearest neighbor joining tree (numbers adjacent to branches are support values from 1000 bootstraps). The tree is drawn to scale in TreeView [26], with branch lengths measured in the number of substitutions per site - 0.1 on the scale bar represents 4 substitutions in 100 bp. The evolutionary history was inferred from 224 aligned characters. The GenBank accession numbers for each stain are shown in parenthesis

The fungus resides in the Eukaryota, in the Fungal Kingdom, and is a member of the Phylum Ascomycota (Table 1). It is considered a member of the Class Dothidiomycetes and Family Venturiaceae. Several other economically important plant pathogens are members of the Dothidiomycetes, including Septoria leaf blotch of wheat (*Zymoseptoria tritici**=**Mycosphaerella**graminicola*), rice blast, (*Magnaprthe grisea*) and apple scab (*Venturia inaequalis*). *F. effusum* has been classified based on its host range, morphology and molecular characteristics (particularly the cytochrome b [23] and ITS region [24]). In the current report, the phylogenetic relationship of *F. effusum* to other Ascomycota species based on the 18S rRNA genes shows that it is most closely related to members of the family Venturiaceae, genera *Fusicladium* and *Venturia* (Fig. 2). The 18S rRNA gene was located on contig0312 and a 224 bp portion aligned with the sequences from the other fungi was used for the analysis. The phylogenetic analysis was performed using nearest neighbor joining method in CLUSTALX2 [25] with node values based on 1000 replicates. The phylogenetic tree was drawn by TreeView [26]. Members from other genera in the Dothidiomycetes (in which the family Venturiaceae resides) were included as outgroups.

## Genome sequencing information

### Genome project history

The genome of *F. effusum* described here was sequenced in 2011 at the ICBR core facility of the University of Florida, Gainesville, Florida, US. The genome was assembled and annotated at the bioinformatics unit at the same location. The project is deposited in Genbank under Bioproject ID PRJNA285422, and the draft assembly and annotation of the isolate of *F. effusum* described in this article is deposited in the same location. The project data is summarized in Table 2. The project information is in compliance with MIGS version 2.0 [27].

**Table 2:** Project information

MIGS ID	Property	Term
MIGS-31	Finishing quality	High quality draft
MIGS-28	Libraries used	454: paired end sequences with 450b insert; Illumina: 1 kb paired-end library
MIGS-29	Sequencing platforms	Illumina Genome Analyzer IIx/454 GS-FLX Titanium
MIGS-31.2	Fold coverage	170×
MIGS-30	Assemblers	ABySS V1.2.6/Newbler V2.3/Phrap/Paracel Transcript Assembler V3.0.0
MIGS-32	Gene calling method	FGENESB/COG/KEGG (Also BLAST search (NCBI tblastx) against the NCBI NR (non-redundant) database and the genome sequences of *Phaeosphaeria nodorum*, *Pyrenophora teres*, and *Saccharomyces cerevisiae*)
	Locus tag	Locus Tags not reported
	Genbank ID	GDCP00000000
	Genbank date of release	2015-06-01
	GOLD ID	Not established in GOLD
	BIOPROJECT	PRJNA285422
MIGS-31	Source material identifier	Not reported
	Project relevance	Biotechnology/mycology/disease control

### Growth conditions and genomic DNA preparation

The isolate of *F. effusum* was cultured on antibiotic-amended potato dextrose agar (amended as for WA, described above) and incubated for 3 weeks at 25 °C (12 h light/12 h dark), at which time the DNA was extracted from the sample using a ZymoResearch DNA extraction kit (ZymoResearch, Irvine, CA), following a slightly modified protocol for DNA extraction from fungi [23]. A Fastprep FP120 (Savant Instruments, Holbrook, NY) was used to lyse the mycelium. Once obtained, the DNA was quantified using a Nanodrop spectrophotometer (Nanodrop Products, Wilmington, DE) and stored in TE buffer at −20 °C.

### Genome sequencing and assembly

The genome was sequenced using 454 GS-FLX Titanium and Illumina Genome Analyzer IIx sequencing platforms. Two stages of assembly were performed to ensure the accuracy and quality of the contigs. The 454 reads were cleaned by masking repeats and removing primers and/or adaptors used in library preparation. The Illumina reads were cleaned using ‘cross_match’ in Phrap [28] and the cleanup module in PTA V3.0.0 [29] to remove low-quality (<20 phred-like score) and short (<40 bp) reads. These cleaned Illumina reads were assembled with multiple trials (a series of k-mers from 30 bp to 75 bp) using ABySS V1.2.6 [30]. The assembled contigs with ≥2,000 bp from ABySS were computationally chopped into 800-bp fragments (with 200 bp overlapping between two adjacent fragments) and further assembled with the cleaned 454 reads using Newbler V2.3 (454 Life Science), to generate the final contigs and scaffolds. A total of 11,959 contigs and 545 scaffolds (average size = 74.4 Kb; total size = 40.6 Mb) were assembled from over 69.2 million clean reads (7.1 Gb). The largest scaffold was >1.1 Mb. There were 3,113 large contigs (≥500 bp), totaling >40.1 Mb which is typical for a genome in the Ascomycota (36.9 Mb, [31]), and not dissimilar to that reported for *V. pirina* [16] and *V. inaequalis* [32]. The 170× genome coverage indicated that ≥95 % of the genome (42.6 Mb) was covered, based on a comparison of reads from high-quality genome sequences [33].

### Genome annotation

*Ab initio* gene prediction with the FGENESB package (Softberry Inc.) predicted 50,192 ORFs from the 3,113 large contigs, including 18,501 RNA ORFs (36.9 %), which was substantially higher than might be expected for this type of organism. For example, only 6,299 peptides were predicted in the genome of *V. pirina* [34], and 13,233 genes in that of *V. inaequalis* [32]. The draft genome sequence of *F. effusum* was somewhat fragmented and an elevated count of small contigs (a total of 11,959) likely led to prediction of multiple ORFs from some genes that were divided among different contigs. Thus the gene count prediction of this draft genome is tentative. To obtain a more accurate perspective on the functional genes [35, 36], we further annotated the ORFs through BLAST at 1e-4 to three genomes, (*Phaeosphaeria**nodorum*, *Pyrenophora teres*, and *Saccharomyces cerevisiae*), in which only 13,897 ORFs were identified. We also used BLAST against three generic genomic databases (NCBI nr, COG and KEGG), in which there was a total of 18,139 hits. At this less stringent e-value, both numbers are only slightly higher than might be expected in a fungal genome; therefore we conclude that the ORFs identified are likely representative of the functional genes in *F. effusum*.

## Genome properties

The draft genome sequence was based on an assembly of 545 scaffolds amounting to 40,096,772 bp, with a G + C content of 48 %. Of the total predicted ORFs, 17,935 had hits in the nr database, 5,263 were assigned to COGs (12.1 %), and 1,580 ORFs in KEGG databases, respectively. It appeared the predicted number of ORFs by FGENESB was not in an expected range of gene numbers predicted in other fungal genomes. Checking known genes, some were incorrectly predicted into multiple ORFs by FGENESB (data not shown). On the other hand, some of the ORFs without hits in the nr database might not be new functional genes. Transcriptome sequences of the genome could be used to improve the *ab initio* gene prediction in the future. These and other properties of the *F. effusum* genome are summarized in Table 3. The distribution of genes into COG functional categories is presented in Table 4. Of the 5,263 proteins, the most abundant COG category was "General function prediction only" (862 proteins) followed by "Carbohydrate transport and metabolism” (658 proteins), "Amino acid transport and metabolism" (468 proteins), "Lipid transport and metabolism" (364 proteins), “Translocation, ribosomal structure and biogenesis” (323), and "Energy production and conversion" (308 proteins).

**Table 3:** Nucleotide and gene count levels of the genome

Attribute	Genome (total)	
	Value	% of total^a^
Genome size (Mbp)	~40.1	
DNA coding (bp)	23932944(/40096772)	59.7
DNA G + C content (bp)	19203566(/40096772)	48
DNA scaffolds	545	
Total genes^a^	50192	
Protein coding genes	18501(/50192)	36.9
RNA genes	Not reported	
Pseudo genes	Not reported	
Genes in internal clusters	Not reported	
Genes with function prediction	Not reported	
Genes assigned to COGs	5263 (/50192)	10.5
Genes with Pfam domains	Not reported	
Genes with signal peptides	Not reported	
Genes with transmembrane helices	Not reported	
CRISPR repeats	Not reported	

^a^The total is based on the total number of predicted protein coding genes in the annotated genome using FGENESB

**Table 4:** Number of genes associated with general COG functional categories

Code	Value	% age	Description
J	323	6.14	Translation, ribosomal structure and biogenesis
A	27	0.51	RNA processing and modification
K	175	3.33	Transcription
L	265	5.04	Replication, recombination and repair
B	39	0.74	Chromatin structure and dynamics; K Transcription
D	70	1.33	Cell cycle control, cell division, chromosome partitioning
V	74	1.41	Defense mechanisms
T	142	2.70	Signal transduction mechanisms
M	131	2.49	Cell wall/membrane/envelope biogenesis
N	7	0.13	Cell motility; T Signal transduction mechanisms
U	87	1.65	Intracellular trafficking, secretion, and vesicular transport
O	353	6.71	Posttranslational modification, protein turnover, chaperones
C	308	5.85	Energy production and conversion
G	658	12.50	Carbohydrate transport and metabolism
E	468	8.89	Amino acid transport and metabolism
F	129	2.45	Nucleotide transport and metabolism
H	143	2.72	Coenzyme transport and metabolism
I	364	6.92	Lipid transport and metabolism
P	213	4.05	Inorganic ion transport and metabolism
Q	207	3.93	Secondary metabolites biosynthesis, transport and catabolism;
R	862	16.38	General function prediction only
S	156	2.96	Function unknown
-	-	-	Not in COGS

The total is based on the total number of protein coding genes in the genome

## Insights from the genome sequence

The genome provides a useful resource for identifying genes of interest in *F. effusum*. Several genes of interest were annotated, including many from the family of P450 genes (of specific interest are the full-length CYP51A (contig 00394) and CYP51B (contig 00058) genes, which are identified in the genome and may be involved in resistance to the dimethyl inhibitors (DMIs) fungicides). Evidence of the mating type gene was also found (putatively MAT-2, Contig 00032), which will be useful as *F. effusum* is currently known only by its asexual stage (conidia), so mating type gene identification can pave the way to establishing existence of a sexual stage. An analysis has also demonstrated that the genome is a rich resource to obtain microsatellite markers with different motif characteristics for studies of pathogen diversity, and to develop as markers for other genetic studies. Furthermore, the phylogenetic analysis presented confirms the close relationship of *F. effusum* to other members of the Venturiacae and previous observations on the taxonomic relationships among these members of the Ascomycota.

## Conclusions

The annotated ORFs may represent partial or full lengths of most functional genes in the *F. effusum* genome and can be used as a new resource for developing molecular markers for genetic diversity studies, and for other research in biology, ecology and phylogenetics, and for research into host/pathogen coevolution.
